# Dataset of exceptional women directors and carbon information disclosures of global energy companies

**DOI:** 10.1016/j.dib.2023.109650

**Published:** 2023-10-05

**Authors:** Nurshahirah Abd Majid, Amar Hisham Jaaffar, Jeniboy Kimpah

**Affiliations:** a College of Graduate Studies, Universiti Tenaga Nasional, Jalan IKRAM-UNITEN 43000, Kajang, Selangor, Malaysia; b Institute of Energy Policy and Research, Universiti Tenaga Nasional, Jalan IKRAM-UNITEN 43000, Kajang, Selangor, Malaysia; c Optentia Research Unit, North-West University, 1900, Vanderbijlpark, South Africa

**Keywords:** Exceptional women directors, Leadership, Governance, Carbon, Disclosures, Energy sector, Reporting

## Abstract

Dataset in this article contains solid information on the novel dataset of exceptional women directors (EWDs) and carbon information disclosures (CID) of global energy leading companies. The data presented is related to the research article entitled “The Effect of Women's Leadership on Carbon Disclosure by the Top 100 Global Energy Leaders” [1]. In exploring the degree of EWDs’ and the level of CID, the content analysis technique based on the enhanced scoring indicators were deployed to obtain the data of the 97 companies based on accessible materials from companies’ websites or any associated reports such as sustainability, environmental, annual, or integrated reports within three-year periods (2018 – 2020) for the global energy leading companies which listed in Thomson Reuters Database 2017. Data on EWDs’ engagement and CID are extracted from the available information, reports, and materials. The data are collected based on the enhanced version of the EWD's indicators adapted from Ramon Llorens, García-Meca and Pucheta-Martínez [2], Hillman, Cannella and Paetzold [3], and Jaaffar and Amran [4]. Meanwhile, data collected for CID by using Carbon Disclosures Index (CDI) comprising 90 scores within nine aspects; Strategy and Policy; Climate Change Risks and Opportunities; Corporate GHG Emissions Targets; Company Wide Carbon Footprint; GHG Emissions Change Over Time; Energy-Related Reporting; Emission Reduction Initiatives Implementation; Carbon Emission Accountability; and Quality of Disclosure as suggested by the prior scholars [5], [6], [7]. This dataset shed light as an indicator to measure exceptional women director in the energy companies in promoting transparent carbon disclosure performance as well as boosting women leadership involvement and participation in the most polluting sector which aligned with the United Nations Sustainable Development Goals; SDG 5 gender equality, SDG 10 reduced inequality, and SDG 13 climate action.

Specifications Table

**Table:** 

Subject	Renewable Energy, Sustainability, and the Environment
Specific subject area	Exceptional Women Director, Corporate governance, women leadership, carbon reporting, climate change and energy industry.
Type of data	Secondary Data Enhanced Aggregated Score of Exceptional Women Directors on Board Table (Excel file) Figure
How the data were acquired	Data gathered and downloaded manually from the companies’ official websites or any associated reports such as sustainability, environmental, annual, or integrated reports by using Women Directors’ Specifications [2,^3^,8] and Carbon Disclosures Index [5], [6], [7] for each company.
Data format	Analyzed Filtered
Description of data collection	Content analysis of 97 companies’ websites or any associated reports for three years observations (2018 – 2020) with a total 291 observations. The content analysis is employed together with the scoring methodology towards the data based on indices, indicators, categories and classifications from the existing literatures and taxonomies. This data extracted and analyzed by using SPSS statistical software from the content analysis procedure applied.
Data source location	Thomson Reuters Database 2017, companies’ websites, and related reports.
Data accessibility	Repository name: Mendeley Data Data identification number: https://doi.org/10.17632/d2s9yz65mm.4 Direct URL to data: https://data.mendeley.com/datasets/d2s9yz65mm/4
Related research article	Abd Majid, N., & Jaaffar, A. H. (2023). The Effect of Women's Leadership on Carbon Disclosure by the Top 100 Global Energy Leaders. *Sustainability, 15*(11), 8491. https://doi.org/10.3390/su15118491

## 1 Value of the Data

• The data provides insight on the Exceptional Women Director (EWD) score and Carbon Information Disclosures (CID) score of global energy leading companies from the year of 2018–2020.
• The data in this article can aid researchers, companies, stakeholders, and policy makers to assess, quantify, implement, enhance, and compare the extent of Exceptional Women Director (EWD) score and Carbon Information Disclosures (CID) score with other carbon intensive industries.
• The data allows other academicians and researchers to investigate on additional factors that can influences the nexus between of Exceptional Women Director (EWD) score and Carbon Information Disclosures (CID) score in the energy sector or other carbon intensive industries by using the extended version of the statistical analysis such as advanced panel data analysis via content analysis technique based on the scoring criteria (indicator) recommended by this data article.
• Due to the nature of the data, it can be used as one of the indicators that can be correlate or regress with the firm's sustainability performance metrics, and it can also be a dashboard for the firms, implying a tool of communication and quality of information towards stakeholders who compelled firms to endorse the sustainability of humanity, the world, and the community.
• The data is useful for researchers exploring women's inclusion in renewable energy, energy transition, climate resilience, gender equality, and net-zero emissions in the corporate boardroom in order to build a more sustainable future.
• The data can be used as indicators of individual differences in WDs' unique knowledge, expertise, skills, connections, and experience as the corporate governance mechanism which sustained competitive advantages on firms’ short- and long-term sustainability and financial performance in the carbon-intensive and male-dominated sector where women leaders are scarce.
• The data also helpful to generate insightful results for energy relevant agencies such as International Energy Agency (IEA), International Renewable Energy Agency (IRENA), and The Organization for Economic Co-operation and Development (OECD) in reducing gender gap and promotes gender equality in energy related industry.

## 2 Objective

The global climate crisis made corporations, especially the energy industry, accountable for their emissions. In response to United Nations Sustainable Development Goals 2030 [9], companies abundantly disclose information representing sustainable activities, policies, and terms through voluntary corporate reporting to promote climate resilience and empower sustainability without a strong reporting framework that may jeopardize information quality in disclosure practices. However, it turns out to have a poor environmental disclosure and transparency information in various reporting mediums due to unregulated and voluntary nature [10], [11], [12]. To fill the void, this dataset created an industry-agnostic CID index by aggregating metrics and indicators of climate-related disclosure, risks, strategies, and opportunities from all relevant national recommendations, frameworks, and guidelines [5], [6], [7] to prevent companies from greenwashing or exaggerating their CID quality and performance [13]. On the other hands, EWDs' engagement and energy-related experiences should be included in increasing disclosures with better-quality information, especially on environmental issues, to improve firm performance and innovation [14]. Energy is a male-dominated industry, so women's participation is always biased. Thus, this dataset is helpful for the regulations and legal authorities in encouraging women's participation, especially in high-polluting industries like the energy sector. Additionally, women in higher positions and who have diverse expertise and experience, especially in energy-related companies, tend to improve firms' governance and ethical dimension [15,16] especially promoting CID [16]. Even with WD representation with other industry differences, multiple directorships, experiences, background, external connections, and professional expertise can influence their perspectives and viewpoints in enhancing CID, WDs participations in the energy sector remain a problem [17]. Therefore, by using this dataset, it may encourage the regulators and governments to emphasize the policy of WDs' involvement in corporate world as little enforcement and low law abiding especially in emerging economies [15] including in the energy sector. The dataset also may improve the gender diversity and carbon emissions literature as they are scarce and contradictory [18], [19], [20], [21], [22].

## 3 Data Description

The repository [23] comprehends three excel files which indicated that Table A, B and C where each of them are referring to data collected; and derived from the related research article [1]. The content of the files is summarized in Table 1.

**Table 1:** Overview of the repository's content: data file description.

File name	Name of the data in article	File description
CID Scores (Table A).xlsx	Table A	Binary and total CID scores for each company
WDs Engagement (Table B).xlsx	Table B	Percentage of WDs’ engagement based on their classifications and percentage on the board
EWDs Aggregated Score (Table C).xlsx	Table C	WDs engagement scores marked by aggregated score among the four dimensions of EWDs. CID aggregated score based on 90 item of CID index in research article [1]

The data contains 97 companies with 291 observations from Thomson Reuters listings as in the related research article [1]. The data collected using purposive random sampling approach to select the companies as the sample. As derived from the related research article [1], the original tool to measure the CID originally developed by de Grosbois and Fennell [5], Alrazi et al. [6], and Bae Choi et al. [7]. The selected items of CID indicators from the existing literature to be adapted in this data and they are aggregate all 90 items in total categorized under 9 themes. Meanwhile the adopted version of WDs’ engagement classifications developed by Hillman et al. [3] and Ramon Llorens et al. [2] derived from the related research article [1]. Table A [23] reveals the CID total score for the global energy leading companies during the year 2018 to 2020. Table B [23] represents the EWDs Score measured by the percentage of WDs’ classification. Meanwhile in Table C [23], it consists the EWDs aggregated scores along with the CID aggregated score which indicate either they are in the “weak”, “low”, “medium”, and “high” engagement and disclosures. In the repository database, all materials provided includes companies with their binary score for each item and each sheet in the excel represent years for the Table A, Table B, and Table C [23]. The abovementioned listed data served as strategically-framed index to measure the progress towards environmental governance with the empowerment of women [8].

## 3 Experimental Design, Materials and Methods

The data represents 97 global energy leading companies selected based on purposive sampling technique [24] from Thomson Reuters 2017 which these companies also listed in the Fortunes 500 companies’ listings after excluding missing data and/or materials. According to Thomson Reuters' 2017 listings, only three of a possible one hundred companies are not included in this data due to acquisitions by other companies that are themselves listed in the listings. Therefore, we exclude the related companies to avoid redundant analysis and similar data. The data consists of 291 companies for three-year observations from year 2018 to year 2020.

**Fig. 1:**
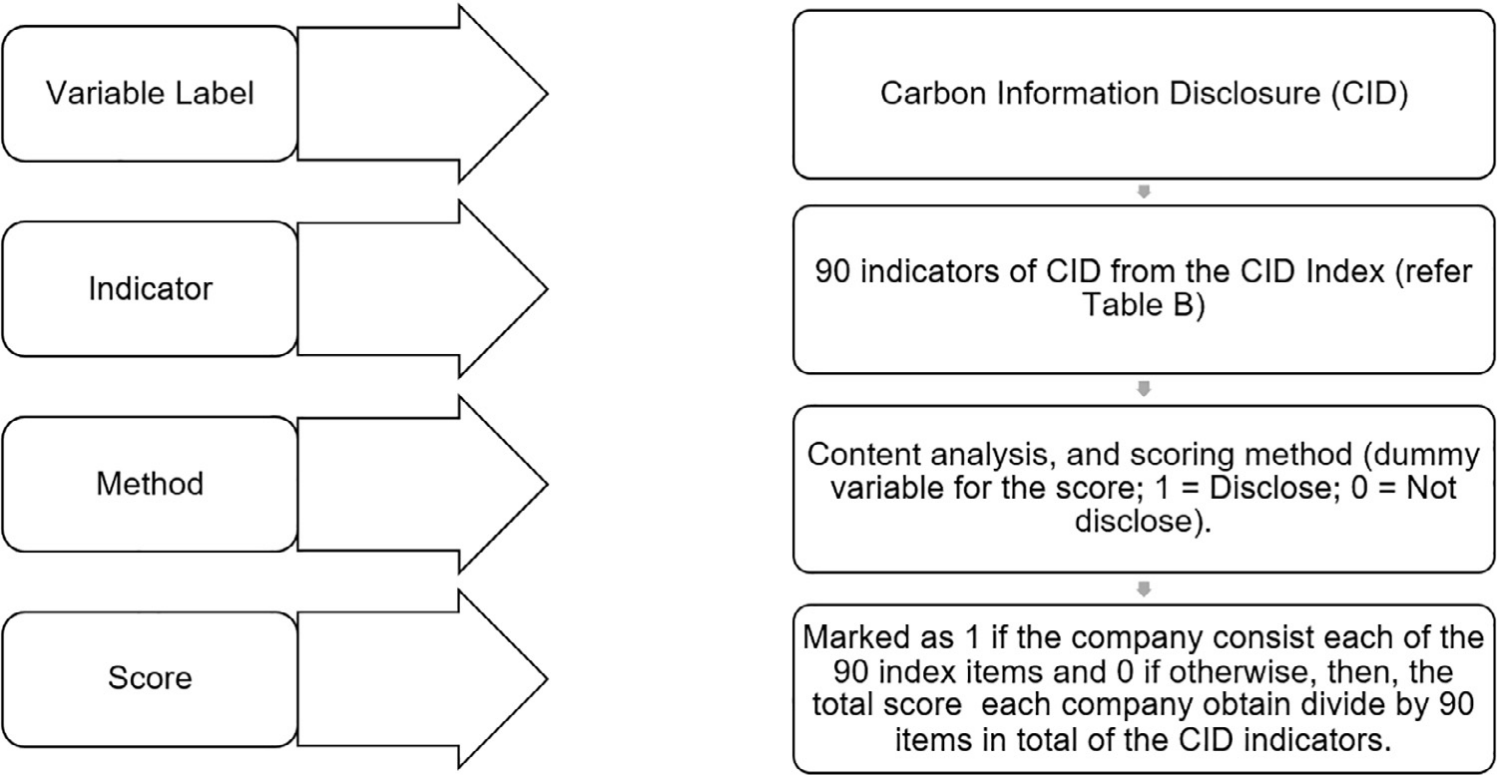
CID dataset collection workflow.

Meanwhile, to compare companies' levels of CID, a hand collected data through a content analysis is used to evaluate and quantified their disclosure to reflect companies' relative commitment of the carbon-related information within four months from November 2021 to February 2022. The content analysis techniques comprise of two techniques such as 1) mechanistic technique (measurement of women in the boardroom or carbon disclosures practices by the number of total words, sentences, summed page proportion, frequency of disclosure and high/low disclosure ratings); and 2) interpretative technique (measurement of women in the boardroom or carbon disclosure practices by qualitative character of the narrative, which focuses on interpretation of text such as the profile/biography of women in the boardroom or firm carbon mitigation strategies in the annual report/company website) [8]. The similar content analysis technique is used to evaluate the EWDs in accordance with their various classifications. Both methods were used simultaneously to gathered the information [25], [26], [27]. By using the content analysis technique, this dataset being collected to identify the CID scores from the index developed by de Grosbois and Fennell [5], Alrazi et al. [6], and Bae Choi et al. [7]; and the number of WD on board along with their engagement criteria which been developed by Ramon Llorens et al. [2] and Hillman et al. [3] and Jaaffar and Amran [4] in order to extract from the sample selection. Meanwhile, the scoring methodology for CID score quantified by awarding one (1) score for the disclosure of CID indicators items in the companies’ websites or any associated carbon-related reports, and zero (0) point if no disclosure is made at all from the basis of the CID index adopted from the prior studies. The data of CID being quantified by the total score of 90 as the overall score from the 9 dimensions of the CID index. The data also consists the CID aggregated score which indicate either they are in the “weak,” “low,” “medium,” and “high” disclosures. Hence, the data collected from this index score will measure how is the level of CID and the more they disclose indicating the more quality of information towards CID may be gathered. Within each of the themes there are items that represent the detailed information of the themes, based on a variety of national reporting; disclosure recommendations and guidelines summarized by existing literatures and scholars [2,^3^,[5], [6], [7] such as Global Reporting Initiatives, Task Force on Climate-related Financial Disclosures (TCFD), Greenhouse Gas Protocol (GHG Protocol), Global Framework for Climate Risk Disclosure (GFCRD), Environmental, Social, and Corporate Governance (ESG), Sustainable Development Goals (SDG), United Nations Global Compact, Sustainability Accounting Standards Board (SASB), Climate Disclosure Standards Board (CDSB), and Carbon Disclosure Project (CDP). In sum, the dataset collection workflow is shown in Fig. 1.

**Fig. 2:**
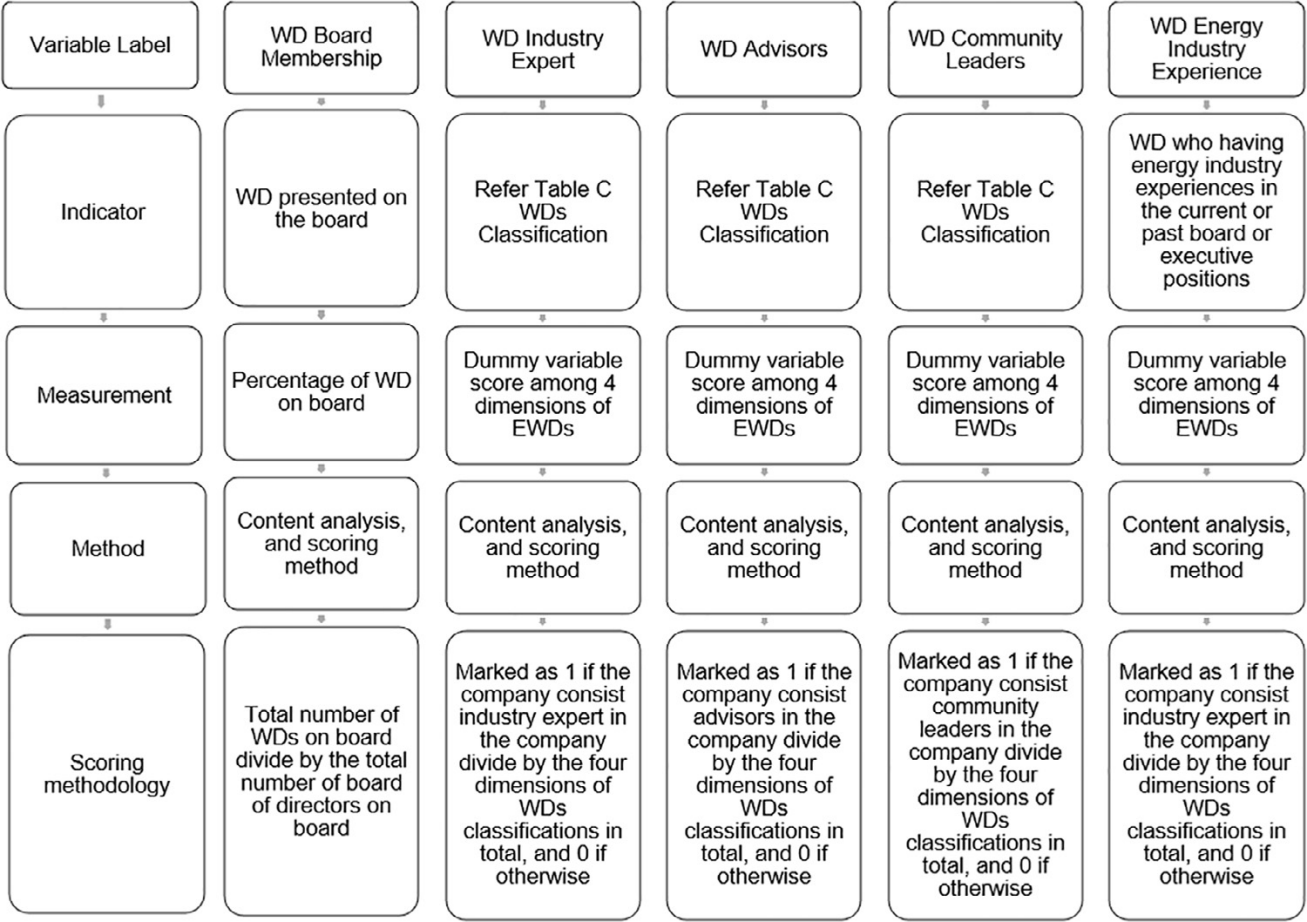
Exceptional women director dataset collection workflow.

In a meantime, the EWDs score indicating the number and percentage of WD from the total directors on board. The EWDs classification from the 4 dimensions are being quantified by scoring one (1) score for having the engagement criterion and zero (0) for not having the engagement criterion at all. It exposing the WDs’ participation on the board among the global energy companies. When identifying and quantifying the data on EWDs criteria, the data can be found that how many and how much the percentages of WDs on the board, what type of classification do the WDs falls under and what they bring into the company; and how these WD classification of criteria will enhance or improves CID among these global energy leading companies. Furthermore, the data consists the EWDs aggregated scores which indicate either they are in the “weak,” “low,” “medium,” and “high” engagement. As the energy sector is the most sector which lack of WDs’ involvement and biased towards their existence in this sector even the WD is very important in promoting CID. The data scoring methodology allows information regarding CID and WD engagement to be examined in depth, their level of disclosure determines the level of quality for the CID information as well as determinations of the WD participation for each company determines the level of WD engagement on board and what expertise, experience, background, knowledge, and external ties they bring into the companies. Therefore, this dataset being collected manually by identifying the number of years of WDs’ energy industry related experiences. In a nutshell, the dataset collection workflow for WDs’ engagement is shown in Fig. 2.

As an overall, after the content analysis technique and scoring methodology process deployed, Table 2 illustrated that more than 59 energy leading companies having a high EWD ranking throughout 3 years. There are at least 9 companies having weak EWD rank. Meanwhile, Table 3 divulges that more than 65 companies out of 97 energy leading companies in a high-ranking CID level from the year 2018 to 2020. Only three companies at the weak CID ranking. Ultimately, all data of each theme of the CID indicators and EWDs’ classifications is provided in supplementary materials (data repository).

**Table 2:** EWD ranking by years.

		Year		
		2018	2019	2020
	
		No. of Companies		
EWDRANK	Weak	10	9	10
	Low	5	4	2
	Medium	21	25	20
	High	61	59	65

*Note:* CID <0–24% = Weak; CID 25–49% = Low; CID 50–74% = Medium; CID 75–100% = High.

**Table 3:** CID ranking by years.

		Year		
		2018	2019	2020
	
		No. of Companies		
CIDRANK	Weak	3	3	3
	Low	2	0	0
	Medium	27	19	12
	High	65	75	82

*Note:* EWD <0–24% = Weak; EWD 25–49% = Low; EWD 50–74% = Medium; EWD 75–100% = High.

## Ethics Statements

No Ethical concerns are involved in the data collection, because the data has been collected from the accessible materials and information on the companies’ websites or any associated reports such as sustainability, environmental, annual, or integrated reports.

## CRediT authorship contribution statement

**Nurshahirah Abd Majid:** Data curation, Formal analysis, Investigation, Resources, Software, Writing – original draft. **Amar Hisham Jaaffar:** Funding acquisition, Methodology, Project administration, Supervision, Writing – review & editing. **Jeniboy Kimpah:** Conceptualization, Visualization, Validation.

## Data Availability

Dataset of Women Directors’ Engagement and Carbon Information Disclosures of Global Energy Companies (Original data) (Mendeley Data)
